# The first direct synthesis of β-unsubstituted *meso*-decamethylcalix[5]pyrrole

**DOI:** 10.3762/bjoc.5.2

**Published:** 2009-01-28

**Authors:** Luis Chacón-García, Lizbeth Chávez, Denisse R Cacho, Josue Altamirano-Hernández

**Affiliations:** 1 Laboratorio de Diseño Molecular, Instituto de Investigaciones Químico Biológicas, Edificio B-1 Ciudad Universitaria CP 58030, Morelia, Mich., México, Fax: +52 443 3265788, Tel: +52 443 3265790

**Keywords:** bismuth, calix[5]pyrrole, calix[*n*]pyrrole

## Abstract

The first direct synthesis of β-unsubstituted *meso*-decamethylcalix[5]pyrrole from pyrrole and acetone, with moderate yield, is described. The results showed that a bismuth salt was necessary to obtain calix[5]pyrrole, with the best results obtained using Bi(NO_3_)_3_.

## Results and Discussion

Calix[*n*]pyrroles have attracted attention because of their ability to recognize anions [1–2]. To date, the calix[4]pyrroles have been studied the most, in part due to the ease with which the macrocycle can be obtained by the condensation of pyrrole with a ketone catalyzed by a Brønsted-Lowry acid such as HCl or methanesulfonic acid, or a Lewis acid such as zeolites with aluminium or cobalt, BF_3_ or a bismuth salt [2–5]. The synthesis of calix[*n*]pyrroles where *n* > 4 has been reported for *n* = 5 or 6. The latter compounds have been synthesized via two routes: a) from the sterically hindered diaryldi(pyrrol-2-yl)methane with 25% yield; and b) through the conversion of a calix[6]furan into the corresponding calix[6]pyrrole by an opening process of the six heterocycles, a selective reduction of the double bond and then a Paal-Knorr condensation with ammonium acetate with 40% yield [6–7]. On the other hand, β-unsubstituted calix[5]pyrroles have been obtained by two routes: a) from the corresponding *meso*-decamethylcalix[5]furan, via a method analogous to that reported for calix[6]pyrroles, with 1% yield; and b) directly when the macrocycle is covalently bound to a calix[5]arene, with 10% yield [8–9]. However, these approaches afford calix[5]pyrroles in low yield, which has limited the study of these compounds as anion receptors.

One explanation for why it is difficult to obtain calix[5]pyrroles via direct condensation of a pyrrole and the corresponding ketone is that the five heterocycle system is unstable: it opens and loses a pyrrole-isopropyl fragment to give the calix[4]pyrrole [8,10].

**Scheme 1:**
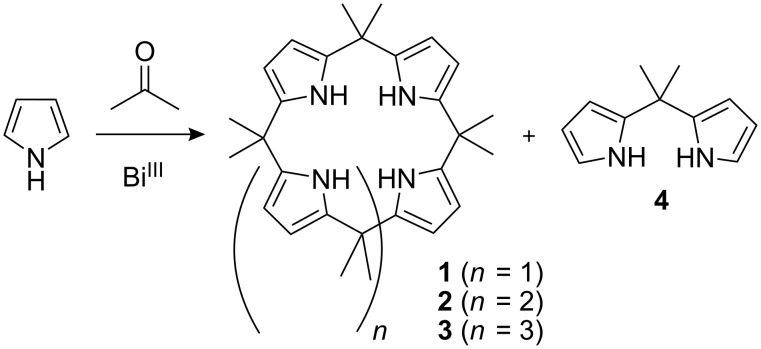
Products obtained by the reaction of pyrrole and acetone with bismuth(III).

In a recent report we described the synthesis of calix[4]pyrroles via the direct condensation of pyrrole with a series of ketones in the presence of a bismuth salt such as Bi(NO_3_)_3_, BiCl_3_, BiI_3_, and Bi(CF_3_SO_3_)_3_, in a 1 : 1 : 0.25 (pyrrole : ketone : BiX_3_) ratio or with the ketone as a solvent at room temperature [5]. Here we describe the first direct synthesis of β-unsubstituted *meso*-decamethylcalix[5]pyrrole (**2**) with Bi(NO_3_)_3_ in moderate yield (Scheme 1).

While studying the role of bismuth as a Lewis acid in the synthesis of calix[4]pyrroles, we found that at low catalyst concentrations some additional products were formed, as observed by ^1^H NMR spectroscopy. These byproducts exhibited ^1^H NMR, ^13^C NMR and MS data consistent with those reported for calix[*n*]pyrroles with *n* = 4, 5 and 6 (compounds **1**–**3**, respectively) and 5,5-dimethyldipyrromethane (**4**); see Experimental section [5–6,8]. The relative proportions of these four products obtained using different catalyst equivalents are listed in Table 1. Compounds **1** and **2** were almost indistinguishable on TLC because of their similar *R_f_* values, and recrystallization from ethanol, as reported in other works, was not satisfactory to give the pure compounds. However, it was possible to separate **1** and **2** by HPLC, to obtain **2** in 25% yield (using the conditions specified in Table 1, entry 12). Compound **2** was found to be unstable, which probably decreased the yield.

**Table 1:** Catalyst conditions and relative proportions of compounds **1**, **2**, **3** and **4** detected in the crude reaction mixture by ^1^H NMR spectroscopy.

Entry	Catalyst	% mol	**1**	**2**	**3**	**4**

1	MgCl_2_ · 6H_2_O	9.5	–	–	–	–
2	CuCl_2_ · 2H_2_O	9.5	100	–	–	–
3	ZnCl_2_	9.5	80	–	–	20
4	AlCl_3_	5	–	–	–	100
5	BiCl_3_	9.5	50	40	10	–
6	BiI_3_	9.5	44	42	12	2
7	BiPO_4_	9.5	53	45	–	2
8	Bi(OTf)_3_	9.5	80	20	–	–
9	Bi(NO_3_)_3_	0.095	–	–	–	100
10	Bi(NO_3_)_3_	0.18	40	–	–	60
11	Bi(NO_3_)_3_	0.32	50	50	–	–
12	Bi(NO_3_)_3_	0.65	33	67	–	–
13	Bi(NO_3_)_3_	0.95	90	10	–	–
14	Bi(NO_3_)_3_	9.5	95	<5	–	–
15^a^	Bi(NO_3_)_3_	25	100	–	–	–

^a^As reported in [5].

To determine whether the reaction proceeds with other Lewis acids, we explored the use of MgCl_2_, CuCl_2_, ZnCl_2_, AlCl_3_, BiCl_3_, BiI_3_, BiPO_4_, Bi(OTf)_3_ and Bi(NO_3_)_3_ under the conditions described above. Except for MgCl_2_, which gave none of the byproducts, all of these Lewis acids catalyzed the reaction to give **1** and/or **4** in amounts ranging from traces to moderate yields. Bismuth salts also produced **3**. The results showed that a bismuth salt was necessary to obtain calix[5]pyrrole **2**, with the best results being obtained with Bi(NO_3_)_3_. The advantages of the method described here—namely that bismuth is relatively non-toxic, the macrocycle is obtained in moderate yield, and the synthesis proceeds without any intermediates—make it the best route to β-unsubstituted *meso*-decamethylcalix[5]pyrrole reported to date.

## Experimental

*meso*-Decamethylcalix[5]pyrrole (**2**). In a typical reaction, 6 mg of Bi(NO_3_)_3_, 2 mL of acetone and 0.09 mL of pyrrole were mixed with stirring at room temperature for 6 h. The reaction mixture was filtered and the solvent evaporated without heat. Reactants were not distilled prior to use and heat was avoided throughout the process. *meso*-Decamethylcalix[5]pyrrole was purified from the crude reaction mixture using an Agilent Technologies HPLC 1200 system equipped with a multiple wavelength detector (G1365D) operating at 350 nm. Purification was performed on an analytical Zorbax Eclipse XDB-C18 column (150 × 4.6 mm, Agilent Tech. Santa Clara, CA, USA). The column temperature was maintained at room temperature and the mobile phases consisted of solvent A (80% MeOH/20% H_2_O) and solvent B (100% EtOAc). Separations were performed by the following solvent gradient: 0 min 20% B, 2.5 min 22.5% B, 20–22.5 min 50% B, 24–26 min 80% B, 31–34 min 100% B, 42–47 min 20% B. All increases of solvent B were linearly programmed. The flow rate was 1 mL/min and the injection volume 20 µL. Yield ca. 25%; mp 208–210 °C; ^1^H NMR (400 MHz, CDCl_3_): 1.51 (s, 30H, CH_3_), 5.77 (d, *J* = 2.8 Hz, 10H, CH), 7.54 (bs, 5H, NH); ^13^C NMR: 29.3 (CH_3_), 35.3 (C(CH_3_)_2_), 102.8 (CH), 138.5 (β-C pyrrole); EIMS *m*/*z*: 535 (M^•+^).
